# Effect of Breast Cancer Surgery on Upper-Limb Muscle Activation

**DOI:** 10.3390/s26072228

**Published:** 2026-04-03

**Authors:** Francisco Franco-López, Alejandro Hernández-Belmonte, Ana María García-Segura, Jaime López-Bueno, Alejandro Martínez-Cava, Javier Courel-Ibáñez, Jesús G. Pallarés

**Affiliations:** 1 Human Performance and Sports Science Laboratory, Faculty of Sport Sciences, University of Murcia, 30720 Murcia, Spain; francisco.francol@um.es (F.F.-L.); anamgarseg@correo.ugr.es (A.M.G.-S.); lobuejai@alumni.uv.es (J.L.-B.); alejandro.martinez12@um.es (A.M.-C.); jgpallares@um.es (J.G.P.); 2 Department of Sports Sciences, Faculty of Medicine, Health and Sports, Universidad Europea de Madrid, 28670 Madrid, Spain; alejandro.hernandez@universidadeuropea.es; 3 Department of Physical Education and Sports, Faculty of Education and Sport Sciences, University of Granada, 52005 Melilla, Spain

**Keywords:** electromyography, asymmetries, velocity loss, strength

## Abstract

This two-step design used the unilateral bench press to examine the effect of breast cancer surgery on upper-limb muscle activation under low and moderate fatigue conditions. First, we studied the proper method to normalize the activation values obtained during dynamic contractions. For that, the muscle activation was relativized to the maximal value obtained during (i) an isometric contraction (ISO_Norm_), and the concentric phase of the (ii) repetition maximum load (1RM_Norm_), and (iii) the first three repetitions of an 80% 1RM set (Max80%_Norm_). The normalization method with the lowest inter-subject variability was further used to compare the muscle activation of the affected and non-affected sides of twelve women who underwent unilateral breast surgery (eight mastectomies and four lumpectomies). Both sides were tested using dynamic sets at 60 and 80% of their 1RM until reaching 40% velocity loss (VL). Repetitions completed at each %1RM were then divided into two groups: low fatigue (first half of repetitions) and moderate fatigue (second half of repetitions). On results, the ISO_Norm_ and the Max80%_Norm_ showed the highest (mean CV = 32.9%) and lowest (mean CV = 12.9%) inter-subject variability, respectively. The affected side showed higher activation for the deltoid and triceps (Δ = 6.9 to 15.9%) but lower for the pectoralis (Δ = −5.7 to −13.2%) against 60% 1RM. These differences were lower and without a consistent trend against 80% 1RM. Between-side comparisons were not significant for either 60% 1RM (*p* > 0.270) or 80% 1RM (*p* > 0.500). Although these results should be interpreted with caution due to the small and heterogeneous sample, our analyses did not reveal meaningful differences in upper-limb muscle activation following breast cancer surgery.

## 1. Introduction

Breast cancer is the most commonly diagnosed malignant neoplasm in women and the leading cause of cancer death for this sex [1]. Advances in breast surgical techniques (e.g., mastectomy, lumpectomy) and therapies (e.g., radiotherapy, chemotherapy) have enhanced survival rates and reduced recurrence [2,3]. Despite this step forward, many patients undergoing these local and systemic treatments experience at least one short-term impairment or disability on the affected side (e.g., lymphedema, pain, or reduced mobility), which may persist for years [4,5]. Among treatments, the local tissue damage caused by surgery, affecting structures such as muscle fascia and nerves, makes it a major contributor to these disabilities. For example, restricted shoulder motion and stiffness are sixfold and threefold more likely, respectively, in patients treated with mastectomy (removal of all breast tissue, sometimes including nearby structures) compared with those who have undergone lumpectomy (removal of the tumor and a small volume of surrounding soft tissue) [6,7]. Moreover, patients undergoing mastectomy are six times more likely to self-report increased difficulty using their upper extremities during activities of daily living [8]. The aforementioned negative effects may worsen 1–2 years post-surgery [9], thus highlighting the need to incorporate post-surgery strategies focused on their prevention or at least mitigation, such as resistance training programs.

Another common imbalance between the affected and non-affected sides is related to strength. Previous studies reported strength deficiency for the affected side, which could persist 10 years post-surgery [10]. Therefore, when access to strength and conditioning coaches is available, they should prioritize first evaluating the strength level of each side and then prescribing unilateral training to accurately tailor the resistance stimulus. Indeed, using bilateral training instead of unilateral training could be behind the reason why strength deficiencies for the affected side persist or even increase after resistance interventions [11]. However, in addition to adapting the resistance training programs, it would be of great value to know the physiological causes behind this common strength deficiency.

Clinical evidence found that the peripheral nervous system of breast cancer survivors is often altered by both mastectomy [12] and breast-conserving [13] surgeries. Therefore, one of the causes behind this strength deficiency of the affected side could be muscle activation. To date, most of the studies on the topic have used isometric contractions to examine the effects of breast cancer surgery on upper-limb muscle activation [14,15,16]. Although isometric contractions allow for a very precise standardization, they are less ecologically valid in relation to daily dynamic movements. On the other hand, studies testing this aspect dynamically have meaningful methodological drawbacks. For instance, they used dynamic bilateral tests [17], which allow for a more stable execution compared with unilateral evaluations but hinder the tailored prescription of the relative intensity (%1RM) to each side due to the aforementioned strength asymmetry (i.e., the same load would represent a different %1RM for each arm). This limitation could be solved by using the load–velocity relationship, which has proven to be valid and accurate to prescribe unilateral %1RM for breast cancer survivors [18]. Moreover, dynamic comparisons to date have been normalized by using maximal isometric values, which are extremely dependent on the joint angulation (not all the muscles tested are in their optimal length–tension relationship) and have lower reliability [19,20,21,22].

These aspects highlight the need to examine the effect of breast cancer surgery on upper-limb muscle activation by unilaterally prescribing dynamic contractions alongside more appropriate normalization procedures. For that, the current research conducted a within-subject design using the unilateral bench press. By using this exercise, we first examined the most reliable normalization method, which was subsequently applied to compare the affected and non-affected sides of women who underwent unilateral breast surgery in terms of muscle activation under low and moderate fatigue levels. We hypothesized that surgical intervention on the affected side would significantly influence muscle activation under both fatigue levels.

## 2. Materials and Methods

### 2.1. Subjects

Twelve women aged 18 to 65 years who had undergone surgery (mastectomy or lumpectomy) after being diagnosed with breast cancer were recruited. Body composition was measured using an electrical bioimpedance (Tanita BC-418, Tanita Corp., Tokyo, Japan), and medical information was obtained through a medical screening (Table 1). Exclusion criteria were: (i) having been diagnosed with another neoplastic pathology or with metastatic breast cancer, (ii) having any illness or mental condition that prevents proper compliance with the procedures (e.g., Alzheimer), (iii) having doctor-confirmed lymphedema and (iv) having any comorbidity that might contraindicate maximum efforts (e.g., decompensated heart failure or uncontrolled high blood pressure). All participants had received their last treatment session (chemotherapy and/or radiotherapy) at least 7 months before participating in the study. The study was conducted according to the Declaration of Helsinki and approved by the Ethics Commission of the Local University. All participants signed a written consent form after being informed of the purpose and experimental procedures. As part of the patient and public involvement approach, two members of the cancer associations from which the women were recruited reviewed the protocol and facilitated recruitment through the support groups.

### 2.2. Study Design

**Figure 1:**
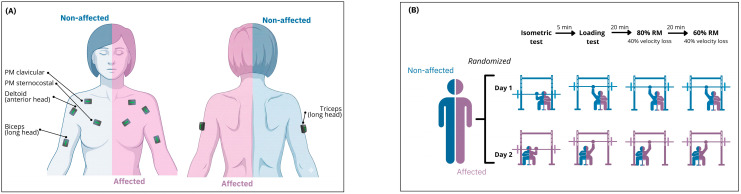
Specific placement of the electromyography sensors (**A**) and study design (**B**). PM: pectoralis major.

This cross-sectional experiment required three visits to the laboratory (one for familiarization and two for testing) (Figure 1). In the second visit (1st testing session), the position of the electromyography sensors was determined on the target side (affected or non-affected; randomly selected) (Figure 1A). After that, an isometric and one-repetition maximum (1RM) test was conducted on the unilateral bench press to obtain the maximal isometric and dynamic activation for each muscle, respectively (Figure 1B). As detailed later, both maximal activation values were compared in terms of inter-subject variability. The load–velocity (L-V) relationship for each side was obtained from the 1RM progressive loading test. After 20 min rest, this individual L-V relationship was used to accurately program the 80 and 60% 1RM (in that order) with which women completed one set of dynamic repetitions until reaching 40% velocity loss (VL). After 72 h of rest, the aforementioned procedures were repeated on the other side in the third session (2nd testing session).

### 2.3. Testing Procedures

#### 2.3.1. Maximal Isometric Activation in the Unilateral Bench Press

The test was performed on a Smith machine (Peroga Fitness, Murcia, Spain) with the subjects placed supine on a bench and their feet positioned on the floor. The isometric position was individually adjusted for each woman by fixing the bar 1 cm above her chest using height-adjustable iron supports. Once this barbell position was determined, it was subsequently used also for dynamic tests (i.e., the beginning of the concentric phase; detailed later). The hand of the target side was placed in the center of the bar, whereas the other was placed on the abdomen. The bench, located on the opposite side of the target side, was carefully adjusted so that the vertical projection of the bar corresponded with each woman’s intermammary line. Besides agreeing with previous unilateral bench press studies [23], this execution position was used after being chosen as the most comfortable for conducting this exercise during pilot studies. During maximal voluntary isometric contractions (MVCs), subjects were instructed to push against the bar as hard as possible for five seconds. Before the first recorded MVC, participants performed a standardized warm-up: one rep at ~50% of the perceived MVC, one rep at ~75% of the perceived MVC, and one rep at 100% of the MVC (3 min of rest between reps). The highest activation value for each muscle after two isometric attempts (separated by a 3 min rest) was considered by further analysis.

#### 2.3.2. Progressive Loading Test

The L-V relationship of the unilateral bench press exercise was determined through a progressive loading test up to the 1RM. The participants’ position and grip were the same as described for the isometric test. A lightweight carbon bar (3.5 kg; patent ID: U202231837) was installed in a Smith machine [24], thus allowing researchers to study a sufficient number of loads up to the 1RM. After measuring the mean propulsive velocity (MPV) [25] attained at the first load, bar weight was gradually increased in 2.5 kg increments until MPV was ~0.42 m·s^−1^ [26]. Then, the weight was individually adjusted in smaller increments of 1 kg until reaching the heaviest load that each woman could properly lift (i.e., 1RM). The participant was considered to have reached her 1RM when the MPV of the last successfully lifted load was ≤0.21 m·s^−1^ [24]. Once the 1RM was determined, the L-V relationship (i.e., the MPV associated with each %1RM) was elaborated accordingly [27]. MPV was recorded using a reproducible and repeatable linear velocity transducer (T-Force System, Ergotech, Murcia, Spain) [28].

#### 2.3.3. Fatigue Test

Twenty minutes after the unilateral bench press progressive loading test, participants conducted two sets of repetitions until reaching 40% VL against 80 and 60% 1RM (in that order). The MPV obtained from individual L-V relationships was used to accurately program the aforementioned intensities (i.e., by adjusting the absolute weight until the MPV of the first repetition of the set matched that expected) [29]. When this absolute load was identified, participants completed a continuous set of repetitions (maximal intended velocity and full range of motion) until reaching 40% VL ([initial velocity − current velocity]/initial velocity · 100). After 20 min of passive recovery, women completed the set of repetitions against 60% 1RM. In each testing session, participants completed the sets against 80% 1RM first, due to the lower metabolic and mechanical fatigue it produces compared with 60% 1RM (when the VL is equaled) [30]. To check a proper recovery of participants before both the first set (after the 1RM test) and the second set of repetitions, two repetitions against the lightest load lifted in the incremental test were measured. If the attained MPV was 0.03 m·s^−1^ lower than that achieved during this incremental test (no fatigued status), the rest was extended by 5 min. The total number of repetitions completed until 40% VL at each %1RM was divided into two groups to examine the activation of each muscle under low (first half of repetitions; 0 to ~20% VL; LOW_fatigue_) and moderate fatigue (second half of repetitions; ~20 to ~40% VL); MOD_fatigue_) levels.

#### 2.3.4. EMG Signal Acquisition

The bipolar Trigno™ sensors (Delsys Inc, Natick, MA, USA) were used to measure surface electromyography (rejection ratio >80 dB, bandwidth filter = 20 to 450 Hz ± 10%). The baseline noise was <5 μV peak-to-peak, and the sampling rate was 2000 Hz. After skin preparation (shaving, sanding and cleaning with alcohol), surface electrodes were placed over the pectoralis major (clavicular portion, PM_Clav_; and sternocostal portion, PM_Ster_), deltoid (anterior head), biceps brachii (long head), and triceps brachii (long head) of the target side (Figure 1A). Electrode positions were located by following the recommendations proposed by the SENIAM project and the surface electromyography protocols described by Criswell [31]. Once the sensors were located, the activation of the aforementioned muscles was measured during both the isometric and dynamic contractions (Figure 1B). The raw activation data was registered using EMGworks^®^ Acquisition software version 4.8 (Delsys Inc, Natick, MA, USA) and subsequently analyzed by using a professional programming code (Sports Science and Human Performance Ltd., Murcia, Spain). The Root Mean Square (RMS; window = 100 ms; overlap = 1 ms) was calculated from raw activation data. Regarding the fatigue test (i.e., dynamic unilateral bench press), only the concentric phase of each repetition was analyzed. For that, the EMGworks^®^ Acquisition software was synchronized with the position data provided by the linear velocity transducer (T-Force). Accordingly, the concentric phase of each repetition was defined as the interval extending from the initial displacement value of zero (i.e., bar 1 cm above the chest, resting on the height-adjustable iron supports) to the final positive displacement value preceding the commencement of the eccentric (downward) phase.

As commented above, the total number of repetitions completed until 40% VL was divided into two fatigue levels: LOW_fatigue_ and MOD_fatigue_. The muscle activation corresponding to each fatigue level resulted from averaging the RMS obtained for the concentric phase of each of the repetitions included in that level.

#### 2.3.5. EMG Normalization Values

To examine the proper normalization reference value to subsequently compare between-side muscle activation, a preliminary analysis was made. For that, the activation of each muscle during the fatigue tests was normalized by considering as a reference value the maximal activation obtained during (i) the isometric contraction (ISO_Norm_) [17], as well as during the concentric phase of the (ii) 1RM load (1RM_Norm_) [32], and (iii) the first three repetitions of the 80% 1RM set (Max80%_Norm_) [20,21]. Only the normalization method with the lowest inter-subject variability was subsequently used for the main between-side comparison.

### 2.4. Statistical Analysis

Before any comparison, outlier values originating from technical artifacts were identified using the robust regression and outliers utility (ROUT) method (Q  =  1%) [33]. Standard statistical methods were used to calculate means and standard deviations (SDs). Normality and homoscedasticity were verified using Shapiro–Wilk and Levene’s tests, respectively. Inter-subject variability of the different normalization methods was calculated using the coefficient of variation (CV; SD/mean · 100) and the relative standard error of the measurement (SEM; computed from the square root of the mean square error term in a repeated-measures ANOVA). Differences between the affected and non-affected sides were examined using paired *t*-tests. To control for the inflation of Type I error rate derived from multiple comparisons at each %1RM (5 muscles × 2 fatigue conditions), *p*-values were adjusted using the Benjamini–Hochberg False Discovery Rate procedure. The effect size (ES) corrected for small sample bias (i.e., Hedges’ g) and the percentage of difference (Δ; calculated as ((affected − non-affected)/non-affected) × 100) were also computed. Analyses were performed using SPSS software version 27.0 (IBM Corp., Armonk, NY, USA) and figures were designed using the Prism GraphPad Prism software (version 9.0).

## 3. Results

The twelve women completed all the sessions included in the study design (no missing sessions). Against 60%, the number of repetitions included in each fatigue level was: LOW_fatigue_ = affected: 7.4 ± 1.7 reps, non-affected: 8.7 ± 2.1 reps; MOD_fatigue_ = affected: 6.6 ± 1.5 reps, non-affected: 8.3 ± 2.1 reps. This number was LOW_fatigue_ = affected: 3.8 ± 0.7 reps, non-affected: 3.6 ± 0.7 reps, MOD_fatigue_ = affected: 3.2 ± 0.7 reps, non-affected: 3.3 ± 0.9 reps, against 80% 1RM.

Table 2 shows the analysis made to study the inter-subject variability for the three normalization methods examined. The ISO_Norm_ and the Max80%_Norm_ showed the highest (Overall CV, affected side = 34.6%, non-affected side = 31.2%) and lowest (Overall CV, affected side = 13.9%, non-affected side = 9.3%) inter-subject variability values, respectively. Therefore, the Max80%_Norm_ method was used for the subsequent between-side comparisons.

**Figure 2:**
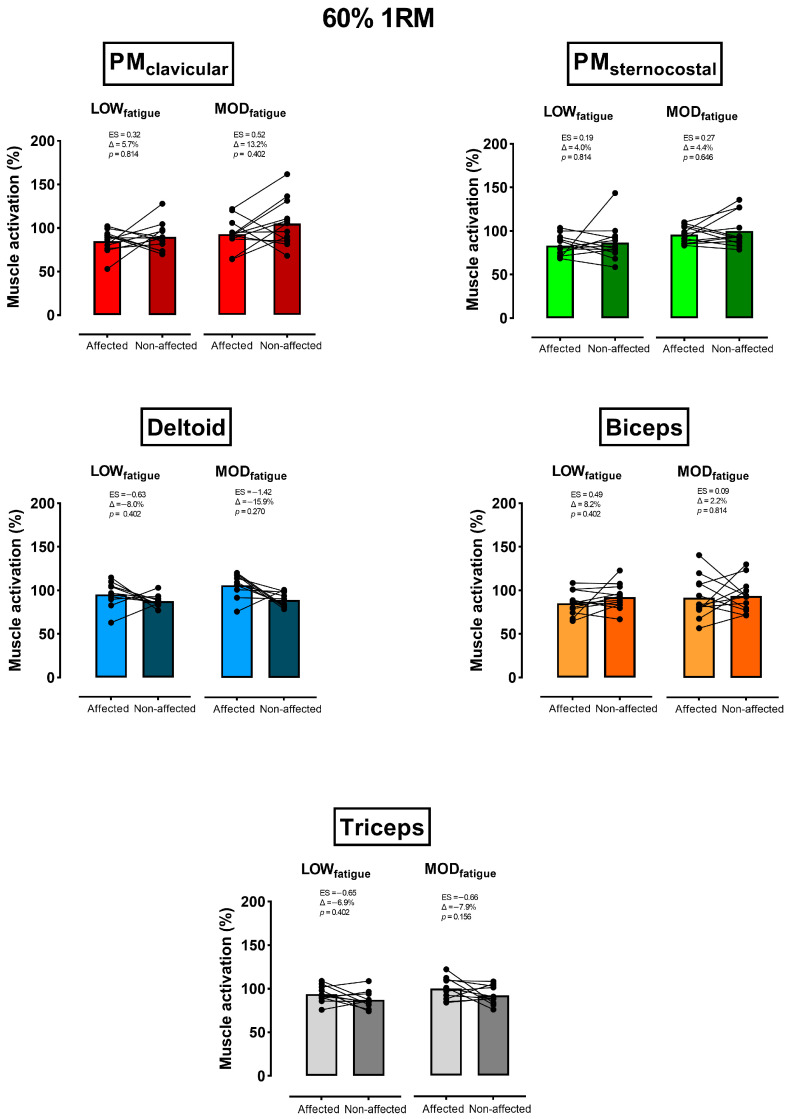
Comparison of the mean (bars; all the subjects) and individual (filled circles and lines) muscle activation between the affected and non-affected sides against 60% 1RM. This within-side comparison was done under low (LOW_fatigue_) and moderate (MOD_fatigue_) fatigue conditions. The *p*-values are shown adjusted by the Benjamini–Hochberg False Discovery Rate procedure.

**Figure 3:**
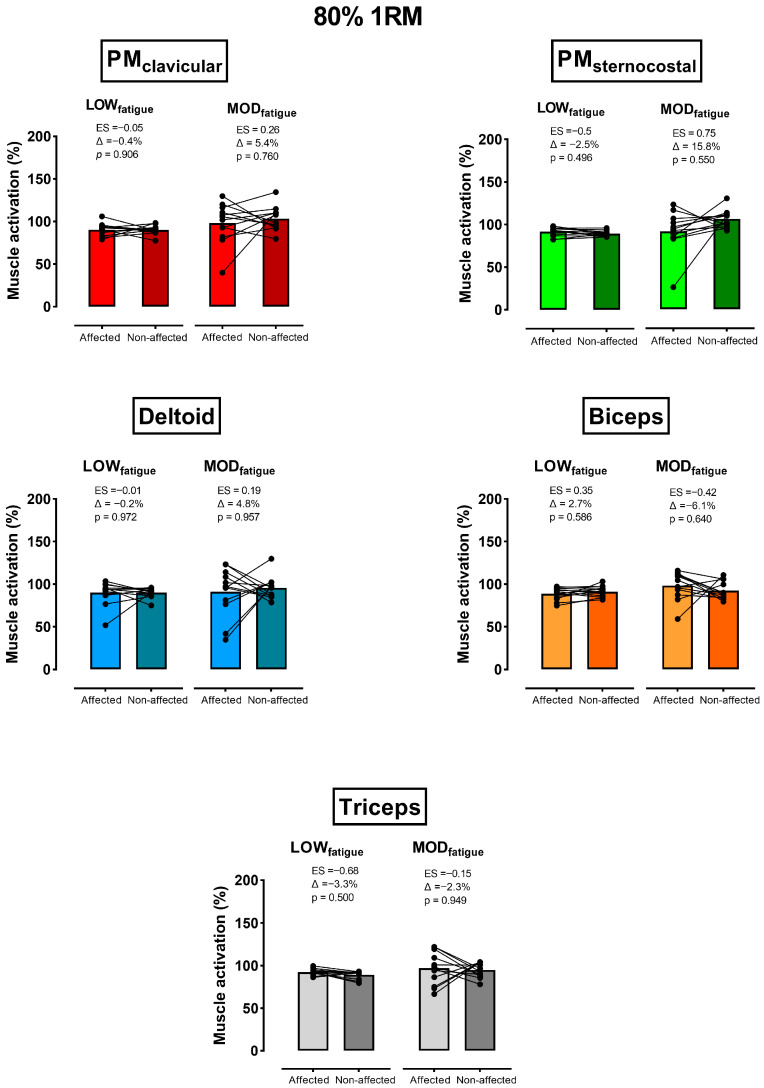
Comparison of the mean (bars; all the subjects) and individual (filled circles and lines) muscle activation between the affected and non-affected sides against 80% 1RM. This within-side comparison was done under low (LOW_fatigue_) and moderate (MOD_fatigue_) fatigue conditions. The *p*-values are shown adjusted by the Benjamini–Hochberg False Discovery Rate procedure.

For both fatigue levels against 60% 1RM, activation was higher for the PM_Clav_, PM_Ster_, and biceps of the non-affected side (Δ = 2.2% to 13.2%), and greater for the deltoid and triceps of the affected one (Δ = 6.9% to 15.9%) (Figure 2). However, no significant differences between sides, either at 60% 1RM (*p* > 0.270) or 80% 1RM (*p* > 0.500), were found. In comparison with sets against 60% 1RM, between-side differences in activation against 80% 1RM (Figure 3) were lower and without a consistent trend among muscles (e.g., non-affected > affected at LOW_fatigue_, and affected > non-affected at MOD_fatigue_).

## 4. Discussion

This two-step design aimed to examine the effect of breast cancer surgery on upper-limb muscle activation under two fatigue conditions. First, we studied the proper method to normalize the electromyography values obtained during dynamic contractions. The normalization method with the lowest inter-subject variability was further used to compare the affected and non-affected sides in terms of muscle activation under low and moderate fatigue levels. Regarding the first step, we found that the Max80%_Norm_ method (i.e., muscle activation during high-intensity but submaximal dynamic contractions) exhibited the lowest inter-subject variability, and therefore, it was used to normalize the subsequent comparisons. On the second step, we only found significantly higher activation for the anterior deltoid of the affected side when reaching moderate fatigue at 60% 1RM. Although these results should be interpreted with caution due to the size and heterogeneity of the sample included, our analyses did not reveal meaningful differences in upper-limb muscle activation following breast cancer surgery.

Although the normalization process is a key step for muscle activation comparisons, there is no scientific consensus on the most adequate method to conduct this step (e.g., isometric vs. dynamic contractions) [19]. Among other characteristics, a normalization method should generate low inter-subject variability [19,22]. On this matter, we found that normalizing muscle activation by maximal dynamic contractions (i.e., 1RM_Norm_) showed lower inter-subject variability than using maximal isometric ones (i.e., ISO_Norm,_ Table 2) [20,21,34]. Isometric contractions provide information on the activation of each muscle at only one specific point of its length–tension relationship, which could not be the optimal one (that at which the tension is maximal) for all the muscles tested due to their intrinsic architecture (e.g., fiber length) [35,36]. Moreover, this specific point of the length–tension relationship is determined by the position of each joint during the isometric contraction [37], which in turn would be highly dependent on the subjects’ anthropometric characteristics (e.g., longer or shorter arms), thus affecting inter-subject variability (Table 2). On the other hand, dynamic contractions would cover the entire length–tension relationship required for each muscle in the specific exercise, thereby increasing the ecological validity of its use as a normalization method, reducing the joint position dependence, and the inter-subject variability. Moreover, we found that this inter-subject variability could be further reduced when using submaximal normalization methods (Max80%_Norm_) [20], probably due to the higher technical stability of these liftings compared with the maximal ones (i.e., 1RM) [38]. In practice, these results together indicate that muscle activation of breast cancer survivors could be normalized throughout submaximal methods, hence reducing the higher fatigue, discomfort, and risk of injury of maximal liftings [27,39].

Although differences between sides were not statistically significant, it is worth noting the considerably higher activation of the anterior deltoid and triceps of the affected side, together with its lower activation of the pectoralis (Figure 2 and Figure 3). Although the current research did not include a force analysis, the aforementioned EMG pattern could complement previous kinetic-based studies proving that synergist muscles (i.e., deltoid and triceps) of the affected side would increase their involvement in torque production to compensate for the lower role of the pectoralis muscles [16]. The fact that the major differences discussed above occurred under moderate fatigue conditions has important training implications. Specifically, coaches implementing the unilateral bench press in breast cancer survivors could be confident that prescribing this exercise using low-fatigue sets (≤20% VL), which in turn have been proven as the most effective and safest intra-set volume to improve strength [39,40,41], would generate similar muscular stimulus between sides.

To the best of our knowledge, this is the first study combining a within-subject design and the velocity-based method to explore muscle activation in breast cancer survivors. Especially referring to the second aspect, addressing the research question throughout the velocity-based method allowed the present study to accurately prescribe the relative intensity and intra-set fatigue for each side [24,27], two key parameters influencing muscle activation and therefore important confounding factors that require exhaustive control. Nevertheless, our research is not exempt from limitations. First, the lack of a control group precludes the current study from knowing whether the non-affected side was actually not influenced by systemic treatments (chemotherapy and hormone therapy) or local therapies with a possible contralateral effect (radiotherapy), or from ruling out the effect of the bilateral asymmetries that are common even in the healthy population. Second, the small sample size included prevented us from performing powerful statistical sub-analyses by subgroups considering differences in the treatment received. For example, the surgery invasiveness (lumpectomy: minimally invasive vs. mastectomy: highly invasive) could be a factor with a significant influence on the activation results. Therefore, our results should be interpreted with caution, considering the heterogeneous and medical conditions of the sample size included (Table 1). Third, the use of surface electromyography did not allow us to study beyond general muscle activation, lacking information provided by more accurate techniques like high-density electromyography (e.g., motor units recruitment or firing rate). Finally, logistical reasons required researchers to test both %1RMs in the same session, so future studies should isolate these evaluations, besides testing lighter and heavier intensities.

## 5. Conclusions

This study did not reveal meaningful differences in upper-limb muscle activation following breast cancer surgery. Future studies including larger patient and healthy (control) samples are warranted to confirm and expand these preliminary findings through subgroup analyses based on patient characteristics.

## Figures and Tables

**Table 1:** Characteristics of the study sample.

Physical Characteristics (Mean ± SD)		
	Age (y)	51.2 ± 10.1
	Weight (kg)	66.4 ± 12.0
	Height (cm)	163 ± 6.0
	BMI (kg/m^2^)	24.9 ± 4.1
	Fat (%)	28.1 ± 7.5
	Lean Mass (kg)	44.6 ± 4.3
	1RM_Rel_ Unilateral Bench Press	0.20 ± 0.05
Medical information *n* (%)		
Disease stage	I	3 (25%)
	IIa	3 (25%)
	IIb	5 (41.7%)
	III	1 (8.3%)
Hormone therapy	Cancer	6 (50%)
	Menopausal symptoms	4 (33.3%)
Treatment received	Chemotherapy	9 (75%)
	Radiotherapy	10 (83.3%)
Surgical procedure	Mastectomy (%)	8 (66.7%)
	Lumpectomy (%)	4 (33.3%)
Axillary surgery	Sentinel Lymph Node Biopsy (%)	2 (16.7%)
	Axillary Lymph Node Dissection (%)	10 (83.3%)
Reconstruction	LD Flap (%)	3 (25%)
	TRAM Flap (%)	2 (16.7%)
Others	Menopausal status (%)	8 (66.7%)

SD, standard deviation; BMI, body mass index; 1RM_Rel_, relative strength ratio (1RM/body mass); LD Flap, Latissimus Dorsi Flap; TRAM Flap, Transverse rectus abdominus myocutaneous; Menopausal status, absence of menstrual periods for 12 consecutive months.

**Table 2:** Inter-subject variation for the different values used to normalize set of repetitions at 60–80% 1RM.

	1RM_Norm_		ISO_Norm_		Max80%_Norm_	
	CV (%)	SEM (%)	CV (%)	SEM (%)	CV (%)	SEM (%)
**Set and side**						
80% 1RM						
Affected	23.5	6.9	32.5	13.8	14.1	3.8
Non-affected	17.5	5.3	32.7	14.1	7.2	2.1
60% 1RM						
Affected	24.7	7	36.7	15.1	13.7	4.4
Non-affected	23.7	7.1	29.7	12.5	11.5	4
**Overall (mean)**						
Overall affected	24.1	6.9	34.6	14.5	13.9	4.1
Overall non-affected	20.8	6.2	31.2	13.3	9.3	3.1

1RM_Norm_: Muscle activation normalized by the maximal value obtained during the 1RM load; ISO_Norm_: Muscle activation normalized by the maximal value obtained during the isometric test; Max80%_Norm_: Muscle activation normalized by the maximal value obtained during the first three reps of the sets against 80%1RM.

## Data Availability

Data are available upon request.
